# SONAR, a nursing activity dataset with inertial sensors

**DOI:** 10.1038/s41597-023-02620-2

**Published:** 2023-10-20

**Authors:** Orhan Konak, Valentin Döring, Tobias Fiedler, Lucas Liebe, Leander Masopust, Kirill Postnov, Franz Sauerwald, Felix Treykorn, Alexander Wischmann, Stefan Kalabakov, Hristijan Gjoreski, Mitja Luštrek, Bert Arnrich

**Affiliations:** 1 University of Potsdam, Digital Engineering Faculty, Digital Health – Connected Healthcare of the Hasso Plattner Institute, Potsdam, 14482 Germany; 2 Ss. Cyril and Methodius University in Skopje, Faculty of Electrical Engineering and Information Technologies, Skopje, 1000 North Macedonia; 3 Jožef Stefan Institute, Department of Intelligent Systems, Ljubljana, SI-1000 Slovenia

**Keywords:** Health care, Scientific data

## Abstract

Accurate and comprehensive nursing documentation is essential to ensure quality patient care. To streamline this process, we present SONAR, a publicly available dataset of nursing activities recorded using inertial sensors in a nursing home. The dataset includes 14 sensor streams, such as acceleration and angular velocity, and 23 activities recorded by 14 caregivers using five sensors for 61.7 hours. The caregivers wore the sensors as they performed their daily tasks, allowing for continuous monitoring of their activities. We additionally provide machine learning models that recognize the nursing activities given the sensor data. In particular, we present benchmarks for three deep learning model architectures and evaluate their performance using different metrics and sensor locations. Our dataset, which can be used for research on sensor-based human activity recognition in real-world settings, has the potential to improve nursing care by providing valuable insights that can identify areas for improvement, facilitate accurate documentation, and tailor care to specific patient conditions.

## Background & Summary

Sensor-based Human Activity Recognition (HAR) is a rapidly growing research field that focuses on identifying and interpreting human movements and behaviors from inertial measurements, such as acceleration and angular velocity. This technology has numerous applications in healthcare, security, and sports analysis^1^. One of the key challenges in HAR is the development of effective algorithms that can accurately and reliably recognize human movements and behaviors in real-world scenarios. To address this challenge, researchers have developed several datasets that capture various types of human activities in different contexts.

Some datasets in the field of sensor-based HAR with Inertial Measurement Units (IMU) are listed in Table 1. Many existing datasets consist of activities of daily living (ADL), such as walking, running, and standing. Collecting data for these activities is relatively easy as they can be performed by many individuals and do not require specialized equipment or settings. As a result, many HAR datasets are focused on these specific activities. It is worth noting that there is also a growing amount of research and datasets focused on more complex activities, such as nursing. Specialized data on nursing activities can provide valuable insights into the nurses’ tasks and help identify areas needing improvement. Analyzing nursing activities can reveal patterns indicating a need for additional training or support. This information can also help nurses tailor their care to specific patient conditions. Moreover, nursing documentation is one factor contributing to an increase in perceived nursing workload^2^. Nurses are estimated to spend between 26.2% to 41% of their time on documentation, which can significantly burden their workload^3^. Classification of the performed activities can facilitate documentation by generating accurate and complete records, thus, reducing errors and omissions. This can then save time and allow nurses to focus on patient care. Overall, using specialized data on nursing activities can enhance the efficiency and effectiveness of nursing care.

**Table 1:** Overview of selected datasets for sensor-based HAR, including sensor type and number, recording time, number of subjects, type of activities, and number of unique activities.

Dataset	Sensor modalities	Recording time in hours	Subjects	Type of activities	Unique activities
OPPORTUNITY^19^	Body-worn sensors: 7 IMUs, 12 3D accelerometers, 4 3D localization information; Object sensors: 12 objects with 3D acceleration and 2D rate of turn; Ambient sensors: 13 switches and 8 3D accelerometers	19.75	4	ADL: groom, relax, prepare/drink coffee, prepare/eat sandwich, cleanup, break, open/close fridge, etc.	35
PAMAP2^20^	3 IMUs and a heart rate monitor	641.75	9	ADL: walking, cycling, playing soccer, ironing, house cleaning, rope jumping, etc.	18
Skoda^21^	20 3D accelerometers	3	1	ADL: write notes, open engine hood, close engine hood, check door gaps, open door, close door, open/close two doors, check trunk gap, open/close trunk, check steering wheel	10
UCI HAR^22^	Smartphone with embedded inertial sensors. 1 waist-mounted smartphone	3.43	30	ADL: walking, walking upstairs, walking downstairs, sitting, standing, laying	6
SHL^23^	5 Body-worn camera and sensors from 4 smartphones	750	3	Locomotion and transportation data: car, bus, train, subway, walk, run, bike, still	8
HARTH^24^	2 3D accelerometers	35.9	22	ADL: sitting, walking, standing, shuffling, transport etc.	12
SONAR	5 IMUs	61.7	14	Nursing activities in a nursing home: washing, change clothes, serve food, etc.	23

Inoue *et al*. conducted a comprehensive study of nursing activities where they collected data under controlled laboratory conditions and real-world settings. It should be noted that this study stands out as a singular and robust dataset in the field of nursing research. When evaluating Inoue *et al*.‘s data collection methods, several factors should be taken into account:

1. The data was primarily collected in a hospital environment, potentially limiting its representativeness for other healthcare settings^4^. Furthermore, the unavailability of the data to the public could restrict accessibility and hinder further research or analysis.
2. The data was collected under controlled laboratory conditions, which might not accurately reflect the actual working conditions of nurses and may fail to capture the full spectrum of factors influencing their performance^5^.
3. The data derived from a larger study^6^ was published in segments to facilitate multiple competitions aimed at determining daily nurse care activities^7–9^. This approach may limit its utility for broader research purposes and pose challenges for comprehensive data analysis.

SONAR (Sensor-Oriented Nursing Activity Recognition) differs from other datasets in several ways.

- The dataset was collected in a real-world setting at an elderly care facility, where experienced professional nurses (spanning between the ages 24–59 and representing a mix of genders) provided care to residents who relied on assistance due to physical or mental limitations. These residents, no longer capable of living independently, resided in single rooms.
- It encompasses a wide array of nursing activities that reflect the multifaceted responsibilities of healthcare professionals. These activities span from critical tasks such as administering medication, taking vital signs, and assisting patients with mobility to important routines such as hygiene care and documentation. This comprehensive coverage enables the development of algorithms that cater to the intricate and diverse demands of the nursing profession. A detailed description of the recorded nursing activities, corresponding actions, and time allocation is provided in Table 2.

**Table 2:** Overview of nursing activities captured in the dataset: Each row corresponds to a distinct activity, with accompanying details including activity description, total recording time in minutes, number of recordings, and statistical measures of activity duration in seconds.

Activity	Description	Total Mins	Recordings	Mean ± Std (s)
Null activity	Null activities include all activities that are not subject to documentation requirements and to avoid overlapping of subsequent activities: adjust bed, wheelchair modification, assist in walking, make a phone call, work at the computer, corona test, reposition costumer, take to the bathroom, cut nails, give insulin, change catheter, hold arm, shower the costumer	1465.6	2259	43.9 ± 79.4
Change clothes	Incontinence care, put a bandage, patch, change clothes	414.9	487	51.1 ± 52.0
Serve food	Serve drink, serve food	303.8	252	72.3 ± 107.7
Wash in bed	Wash in bed	200.4	165	72.9 ± 60.5
Kitchen preparation	Make coffee, organize supplies, set up equipment	176.5	164	64.6 ± 90.3
Clean up	Tidy/clean the cupboard, clean up, fold laundry, rearrange laundry, empty catheter	167.2	301	33.3 ± 53.0
Make bed	Put clean sheets on a bed, make bed	161.9	210	46.2 ± 48.5
Deliver food	Deliver tray/dishes, distribute dishes	125.4	375	20.1 ± 19.4
Wash at sink	Personal hygiene, skin care, rinse and refresh	116.6	109	64.2 ± 59.7
Push wheelchair	Push trolley, push wheelchair	115.3	403	17.2 ± 13.5
Put food on plate	Put food on plate	99.2	194	30.7 ± 31.9
Collect dishes	Collect dishes	73.0	183	23.9 ± 23.2
Prepare bath	Prepare bath	64.5	117	33.1 ± 21.8
Wheelchair transfer	Assist with sitting down, assist in getting up, wheelchair transfer	45.1	145	18.7 ± 15.8
Put medication	Administering medication to residents	40.1	11	218.9 ± 372.5
Pour drinks	Pour drinks	34.0	114	17.9 ± 15.9
Dental care	Dental care	27.7	23	72.3 ± 51.0
Documentation	Documentation	26.5	48	33.1 ± 28.3
Comb hair	Comb hair	16.4	47	21.0 ± 20.2
Wipe up	Wipe and tidying up the living spaces of the residents	10.1	19	32.0 ± 47.3
Put accessories	Put a bib, put perfume, put accessories	8.4	38	13.2 ± 9.9
Blow-dry	Blow-dry	5.3	1	317.3 ± NaN
Wash hair	Wash hair	2.1	8	15.7 ± 7.2
- The dataset was collected using five sensors placed on each participant’s body as standalone IMUs. This allows for more comprehensive and detailed views of human movements and behaviors. This methodological choice also facilitates the exploration of diverse sensor combinations and optimal placements, thus, yielding outcomes that are both specialized and incite new research questions. Furthermore, integrating multiple sensors allows for analyzing subtle interplays and trade-offs between singular and multiple sensors.
- The data was initially labeled by an external human observer who walked alongside the nurses. To ensure accurate subsequent labeling and protect privacy, we used synchronized pose estimations of the nurse’s body. The pose estimation data was obtained from parallel video conversion.

Overall, SONAR’s combination of real-world, comprehensive sensor data, and detailed labeling make it a valuable resource for researchers in the field of HAR.

## Methods

In this section, we begin by addressing the ethical considerations that guided our research process. Following that, we provide a comprehensive account of our study’s methodology. This includes detailing the technical specifications of the sensors we employed and describing their placement on study participants. We will also discuss the methods used for data collection and annotation. Additionally, we will explore the study’s design and protocol, followed by an overview of the properties of the recorded data.

### Ethics approval

The study was carried out with the utmost regard for the well-being and rights of the participants. Approval for the study was obtained from the University of Potsdam Ethics Committee, under the reference number 51/2021. All participants willingly contributed to the study after providing informed consent, including consent to publish the data. The participants were thanked for their time and effort, and their contributions were greatly appreciated. The data collected from the sensors were treated with the utmost confidentiality, and appropriate measures were taken to ensure the participants’ privacy was protected throughout the study.

### Equipment

We employed Xsens DOT v2 Bluetooth wearable sensors with accompanying straps throughout this study. These sensors are compact and lightweight: dimensions of 36 × 30 × 11 mm and a weight of 10.8 grams. The design enables the attachment of multiple sensors, thereby, enhancing the capability to capture subtle movement patterns. Upon connecting to the mobile phone, the sensors start to measure and record data. The sensors have a recording frequency and a real-time streaming frequency of up to 120 Hz and 60 Hz, respectively. Xsens DOT features up to 6 hours of continuous data measurement. They use a right-handed Cartesian coordinate system, as illustrated in Fig. 2, with x, y, and z axes. The sensors output data in various formats, including Euler angles, quaternions, delta quantities (acceleration and angular velocity), rate quantities (acceleration and angular velocity), high fidelity data, and custom data combinations. The data format can be selected in the Xsens DOT app or in the SDK. For our study, the sensors were connected to a smartphone via Bluetooth 5.0 devices. Five sensors were paired with a Google Pixel 6 phone for data collection. Output data from the sensors included orientation (in quaternions or Euler angles), free acceleration, angular velocity, magnetic field, timestamp, and status.

### Labeling

**Fig. 1:**
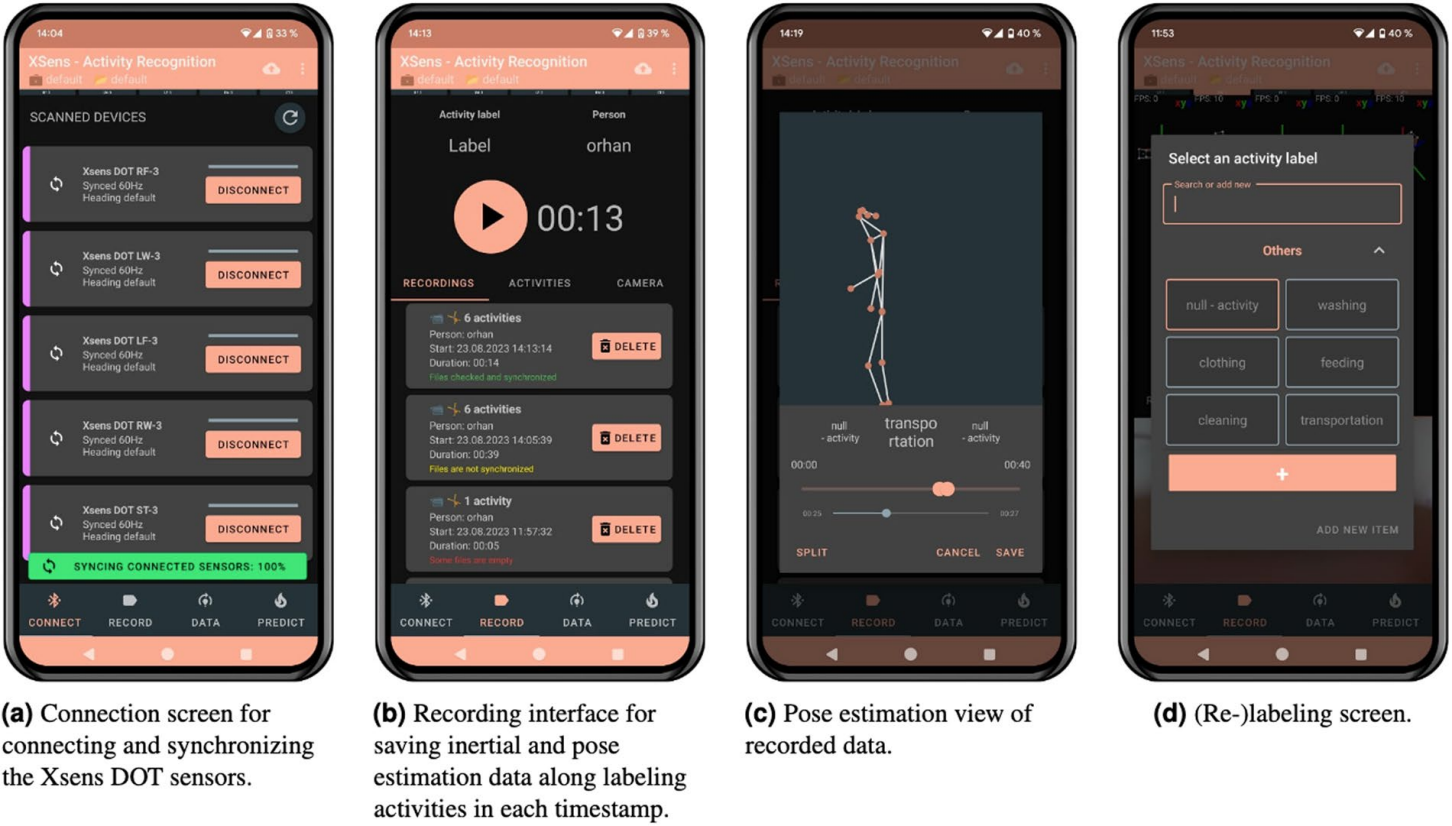
Different functionality screens of the application for data recording.

Our dataset, shown in Table 2, consists of 23 different nursing activities that involve frequent changes in labeling. To address the lack of simultaneous real-time recording and labeling in the Xsens DOT App, we developed our own application^10^, as depicted in Fig. 1. The application not only allows synchronized sensor recordings, but also facilitates real-time pose estimation using a parallel video stream of the nursing activities via the mobile phone’s camera. The outcome is a skeleton model with 17 keypoints of the human body, as shown in Fig. 1c. By converting the video stream into pose estimations, we ensure an accurate relabeling process and secure anonymization where privacy is pivotal, such as in nursing facilities where patients are being washed, dressed, or fed.

Connection screen for connecting and synchronizing the Xsens DOT sensors. Recording interface for saving inertial and pose estimation data along labeling activities in each timestamp. Pose estimation view of recorded data. (Re-)labeling screen.

### Study design

**Fig. 2:**
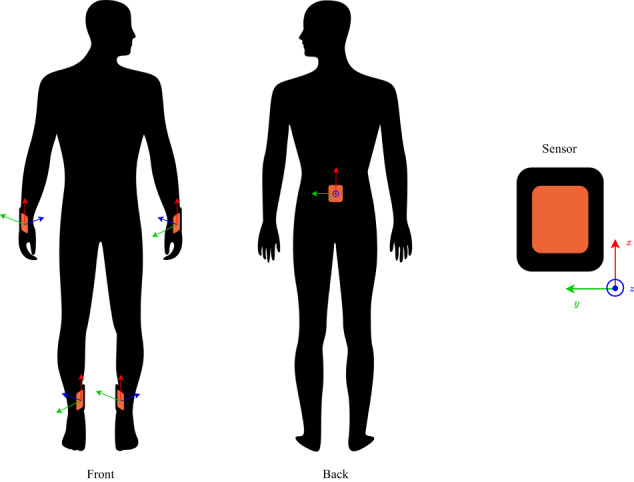
Sensor placement on nurses.

Participants were recruited from a retirement and assisted living facility in Potsdam, Germany. The facility’s primary goal is to provide care and support for elderly people, more specifically, focus on helping them maintain or regain their abilities and skills required for daily life. This is achieved through individualized support which considers each person’s unique needs and circumstances. Prior to data collection, participants received an explanation of the project via mail. Eligibility criteria required individuals to work as nurses. Upon obtaining written informed consent, the nurses were equipped with sensors for data collection. Data was collected using five Xsens DOT sensors with a 60 Hz output rate on the following body locations: left wrist (LW), right wrist (RW), pelvis (ST), left ankle (LF), and right ankle (RF). The sensors LW, RW, LF, and RF were attached to the body by using straps of different lengths with a velcro fastener. The ST sensor was attached on the waistband using a rubber clip. Figure 2 illustrates the placement of the sensors on the nurse. The placement of IMUs was selected based on prior studies demonstrating the effectiveness of these locations in providing robust and accurate information for HAR and pose estimation tasks^11^. The sensors were positioned face down when the character was in an A-pose. In other words, the negative *x*-axis of the character’s pose was aligned with the direction of gravity. The A-pose is a standard reference pose used in animation and computer graphics. In this pose, the character stands upright with its arms extended out to the sides and its palms facing forward, creating a shape that resembles the letter A:

- Outer wrist: The positive *z*-axis is positioned towards the inner wrist, while the negative *x*-axis is positioned towards the hand.
- Pelvis - central on the back waistband: The positive *z*-axis is positioned towards the body, while the positive *x*-axis is positioned towards the head.
- Inner ankle: The positive *z*-axis is positioned towards the outer wrist, while the negative *x*-axis is positioned towards the foot.

After synchronizing the sensors and verifying that the data was being transmitted correctly and completely, nurses were asked to carry out their usual duties while accompanied by an external observer who performed data labeling. The observer recorded the type of nursing activity performed from start to end, and the data was transferred to a mobile device via Bluetooth in real-time. Throughout the study, the observer obtained information from the nurse through verbal communication to ensure that the correct labels were selected. To ensure reliability, each nurse was recorded multiple times performing the same activity on different residents. At the end of each day, the data was transferred to a hard drive and deleted from the phone. Each recording consists of 14 measurements, including, four-dimensional quaternion values, four-dimensional angular velocity calculated from the derivative of the quaternion values, three-dimensional acceleration values, and three-dimensional magnetic field values.

### Dataset properties

A total of 14 nurses, comprising of nine females and five males, aged between 24 and 59 years, participated in the study and provided written informed consent to be recorded while performing their daily work duties. The recording process began with the observer pressing the start button after verifying that the data stream was functioning correctly. One or multiple activities were recorded during each session, and the process ended when the observer pressed the stop button. Throughout the study, 254 recording files with an overall of 5673 recordings were collected; this included 23 different activities that are commonly performed by nurses in the assisted living facility.

The recorded activities were typical nursing duties, such as changing a patient’s clothes or washing their hair and involved similar procedures or movement patterns. These activities were grouped together according to nursing rules and classification criteria aimed to capture the essential elements of each activity for documentary purposes. This approach ensured that the data collected was representative of the different types of activities performed by the nurses and provided a comprehensive overview of the movements involved in each activity.

**Fig. 3:**
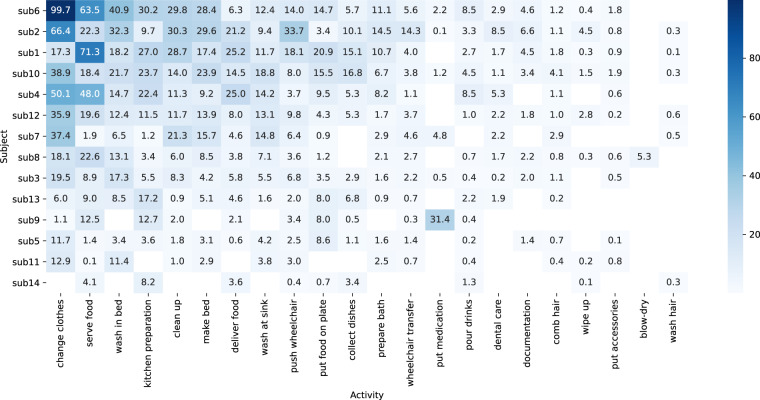
The heatmap visually represents the distribution of data on the number of minutes spent by each subject on various activities. The y-axis displays subjects sorted by their total duration spent across all activities.

**Fig. 4:**
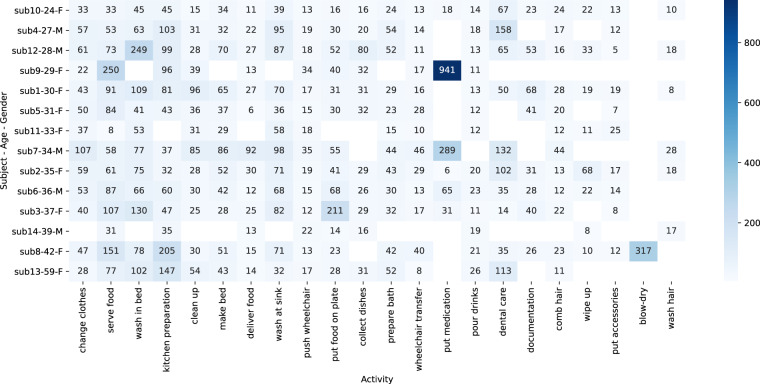
Heatmap Visualization of Average Activity Durations by Subject, Age, and Gender (in seconds): This heatmap illustrates the average duration of various activities recorded by different subjects, with each subject’s age and gender also indicated. The x-axis represents different activities, while the y-axis shows the subjects’ identities along with their corresponding ages and genders, sorted in ascending order by age. The color intensity in each cell reflects the average duration of each activity and subject, providing a visual representation of activity patterns and differences across subjects.

In total, 13319475 data points per sensor stream were recorded, representing approximately 3700 minutes (~61.7 hours) of recording time. Figures 3, 4 and Table 2 display the data distribution among subjects and activities, providing an overview that can serve as a reference for further analysis and comparison with other studies. We employed a systematic approach to ensure accurate labeling and prevent overlapping activities in the recordings. Each recording was either deliberately stopped and categorized as a *null activity* before commencing the next activity, or alternatively, recordings were split based on the subsequently recorded pose estimation (label refinement). This methodology guarantees distinct and precise labeling for each activity.

## Data Records

**Fig. 5:**
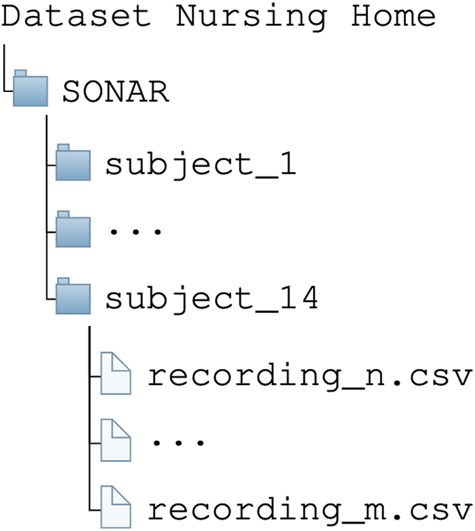
Organization of the SONAR dataset.

The dataset is accessible via Zenodo, allowing easy collaboration and data sharing among researchers^12^. Additionally, there is a preprocessed version of the dataset available, organized into a single folder containing all data records, specifically designed for machine learning purposes^13^. It comprises data gathered from 14 voluntary participants, each providing informed consent to contribute to this study. As shown in Fig. 5, the dataset is organized in 14 folders, one for each participant. Each folder contains the recordings stored as CSV files. The dataset contains recordings from five synchronized Xsens DOT sensors, with each IMU containing 14 measurements. The recordings are stored as CSV files, with each file containing 72 columns. The size of the CSV files varies based on the length of the recording. The first 70 columns correspond to the 14 measurements from the five IMUs, including orientation, acceleration, and calibrated local magnetic field. The two remaining columns in each file contain the timestamp in microseconds and the activity label at each timestamp. The 14 measurements captured by the IMUs are as follows:

- The output orientation is presented as quaternion values, with the real part represented by *Quat_W* and the imaginary parts represented by *Quat_X*, *Quat_Y*, and *Quat_Z* respectively. The name of the sensor is also included in the column names. The Delta_q values represent the orientation change over a specified interval, which is 16.67 ms (60 Hz) for Xsens DOT sensors. The column name is composed of three parts, separated by an underscore: *dq*, indicating the angle increment; *W, X, Y* or *Z*, indicating the real/imaginary part/direction; and the name of the sensor.
- The Delta_v values represent the change in velocity or acceleration over the same interval as the Delta_q values. The column name is divided into two parts, separated by an underscore: *dv*, indicating the velocity increment; and a number between 1 and 3 in square brackets, denoting the axis (1 corresponds to *X*, 2 corresponds to *Y* and 3 corresponds to *Z*). The name of the sensor is also included in the column name.
- The next three columns contain the calibrated local magnetic field in the *X*, *Y*, and *Z* directions, respectively, and are denoted as *Mag_X, Mag_Y*, and *Mag_Z*, followed by the name of the sensor
- The sequence of column names is repeated for all five sensors. The penultimate column, *SampleTimeFine*, displays the timestamp in microseconds.
- The final column contains the label for the activity performed at each timestamp.

## Technical Validation

In this section, we focus on validating the quality and soundness of the dataset deposited at Zenodo by using three different deep learning models as tools to demonstrate the fitness of the dataset for activity recognition tasks. The models were trained and evaluated using the dataset, and their performance serves as evidence of the dataset’s reliability and suitability for further research.

### Data preprocessing

Preprocessing raw data is an important initial step in the deep learning workflow as it prepares the data in a format that can be easily understood and processed by the network. In this study, the following preprocessing steps were applied to the raw data in order to eliminate any unwanted distortions and improve specific qualities.

1. **Imputation**: Missing data values can be a major issue in real-world datasets, making it difficult to effectively train the network. In this study, the missing data values were handled through imputation using linear interpolation to fill NaN values.
2. **Standardization**: The dataset consists of 14 different features recorded in different units. To avoid varying scales and distributions issues, the data was standardized prior to being input into the deep learning algorithm. Rescaling of the values involved standardizing the data to have a mean of 0 and a standard deviation of 1.
3. **Windowing**: In order to better understand the relationships between the features, the sensor data was divided into non-overlapping windows of 600 data points (equivalent to 10-second windows at a recording frequency of 60 Hz). This process provides a broader understanding of the underlying activity measured.

### Deep learning architectures

We trained three different deep learning models incorporating a combination of convolutional neural network (CNN)^14^ and long short-term memory (LSTM)^15^ network components or only a CNN. These models were trained using the Adam optimizer with default settings in Tensorflow^16^. Hyperparameter optimization resulted in a learning rate of 1 × 10^−4^ and an input size of 600 × 70. The input size corresponds to a window size of 600 (equal to 10 s with 60 Hz) and 14 features from each sensor (14·5 = 70). All models include a preprocessing step for filling missing values and a batch-normalization layer to standardize the inputs in each feature row. The output of each network is a dense softmax layer for activity classification. The models were trained using the categorical cross-entropy loss function, which is defined as:

$$L=-log\left(\frac{{e}^{{s}_{p}}}{{\sum }_{j}^{C}{e}^{{s}_{j}}}\right)$$

where *C* denotes the set of classes, *s* is the vector of predictions, and *s*_*p*_ is the prediction for the target class. The architecture of the three models is as follows:

1. The **CNN-LSTM** model is composed of six layers. The input layer is followed by two convolutional layers, two LSTM^17^ layers, and the output layer.
2. The **ResNet** model is composed of 11 layers. Next to the input and output layer, it has a repeated sequence of three convolution layer followed by a batch normalization layer and an activation layer^18^.
3. The **DeepConvLSTM** model is composed of eight layers. After the input layer, it is followed by four consecutive convolution layers and two LSTM layers before the softmax classifier^15^.

### Dataset validation

**Fig. 6:**
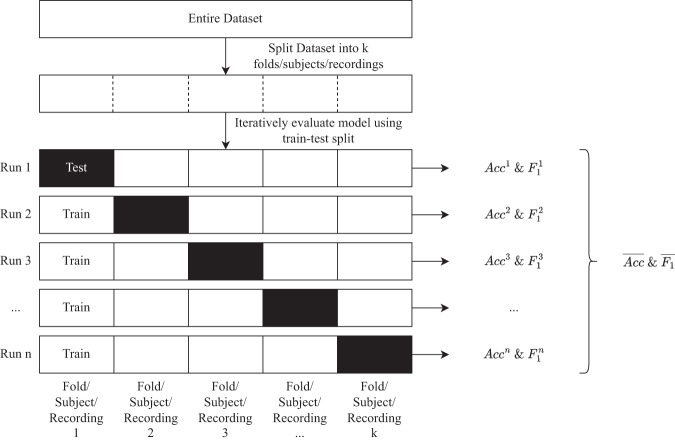
Evaluation strategy.

This section describes the evaluation process, which encompasses model validation methods and performance metrics, collectively contributing to an understanding of the dataset’s reliability. The selection of evaluation metrics depends on the specific machine learning task and is crucial for quantifying the performance of the model. Figure 6 illustrates the evaluation strategy used for the benchmark results. The subsequent subsections delve into the validation techniques and performance metrics in greater detail.

#### Model validation

To state the performance of our models on unseen data, we used the following three different cross-validation techniques:

1. **k-fold cross-validation**: This method divides the entire dataset into *k* equal-length segments, also known as folds. One fold is designated as the test set and is used for final evaluation. In this study, we used five folds, with each fold representing a different time window. This method provides a good balance between having enough data for training and enough data for testing.
2. **leave-recordings-out cross-validation**: This method evaluates the performance of the models on individual recordings. A recording refers to the time frame between pressing the start and stop buttons during activity labeling. In this study, there were 254 recordings, and we used a 80:20 train-test ratio, with 203 recordings for training and 51 recordings for testing. This method evaluates the models’ ability to generalize to new recordings, which is important in this specific task, as recordings can contain a single activity or multiple activities performed multiple times.
3. **leave-one-subject-out cross-validation**: This method involves training the model on all subjects except for one and then evaluating the model on the held-out subject. This process is repeated until each subject has been held out for evaluation once. This method evaluates the models’ ability to generalize to new subjects and maximizes the use of available data. However, it can be time-consuming to train and evaluate the models multiple times.

#### Performance metrics

There are various evaluation metrics that are derived from different ratios of the values true positive (TP), true negative (TN), false positive (FP), and false negative (FN). The following commonly used performance metrics were used for comparison:

$${F}_{1}=2\cdot \frac{Precision\cdot Recall}{Precision+Recall}=\frac{TP}{TP+\frac{1}{2}\left(FP+FN\right)}$$

As shown in Table 2, the dataset is imbalanced with respect to the different classes. To provide greater weight to classes with more examples in the dataset, we chose to use the weighted-averaged *F*_1_ score. This means that the proportion of each label in the dataset is weighted by its size. Additionally, we calculated the micro-averaged *F*_1_ score, which represents the proportion of correctly classified observations out of all observations, also known as accuracy *Acc*, by considering the total number of TPs, FNs and FPs observations.

### Validation results

Tables 3–7 show the weighted-*F*_1_ scores and accuracy alongside the standard deviation in brackets (i) from using different sensor placement combinations, (ii) under different validation setups, and using (iii) three different deep learning models. The best performing model for each sensor and validation setup is highlighted in bold.

**Table 3:** Comparison of different cross-validation methods and different performance metrics (column) for different models on a single sensor (row).

Sensors	Model	k-fold		leave-recordings-out		leave-one-subject-out	
		Acc	*F*_1_	Acc	*F*_1_	Acc	*F*_1_
{LW}	CNN-LSTM	**0.36** (±0.03)	0.31 (±0.04)	**0.36** (±0.04)	**0.3** (±0.03)	**0.36** (±0.04)	0.31 (±0.02)
{LW}	ResNet	0.34 (±0.02)	**0.32** (±0.03)	0.33 (±0.06)	0.3 (±0.07)	0.35 (±0.04)	0.33 (±0.05)
{LW}	DeepConvLSTM	0.3 (±0.02)	0.18 (±0.04)	0.3 (±0.02)	0.19 (±0.02)	0.29 (±0.03)	0.18 (±0.03)
{RW}	CNN-LSTM	**0.35** (±0.04)	0.28 (±0.05)	0.35 (±0.04)	0.28 (±0.05)	**0.36** (±0.03)	0.28 (±0.03)
{RW}	ResNet	0.34 (±0.07)	**0.32** (±0.07)	**0.38** (±0.07)	**0.36** (±0.07)	0.33 (±0.05)	**0.32** (±0.04)
{RW}	DeepConvLSTM	0.27 (±0.02)	0.16 (±0.01)	0.29 (±0.04)	0.16 (±0.04)	0.29 (±0.04)	0.17 (±0.03)
{ST}	CNN-LSTM	**0.32** (±0.04)	**0.25** (±0.03)	**0.3** (±0.03)	0.23 (±0.04)	**0.32** (±0.03)	0.24 (±0.04)
{ST}	ResNet	0.26 (±0.05)	0.24 (±0.05)	0.26 (±0.06)	**0.24** (±0.05)	0.29 (±0.06)	**0.27** (±0.06)
{ST}	DeepConvLSTM	0.28 (±0.06)	0.19 (±0.05)	0.27 (±0.05)	0.16 (±0.05)	0.28 (±0.06)	0.18 (±0.07)
{LF}	CNN-LSTM	**0.29** (±0.04)	**0.23** (±0.06)	**0.29** (±0.03)	**0.23** (±0.04)	**0.29** (±0.02)	**0.23** (±0.03)
{LF}	ResNet	0.21 (±0.03)	0.19 (±0.04)	0.22 (±0.03)	0.2 (±0.03)	0.2 (±0.02)	0.18 (±0.02)
{LF}	DeepConvLSTM	0.24 (±0.03)	0.15 (±0.01)	0.21 (±0.03)	0.13 (±0.02)	0.26 (±0.05)	0.17 (±0.05)
{RF}	CNN-LSTM	**0.32** (±0.02)	**0.25** (±0.03)	**0.3** (±0.04)	**0.24** (±0.05)	**0.29** (±0.04)	**0.23** (±0.04)
{RF}	ResNet	0.23 (±0.06)	0.19 (±0.04)	0.25 (±0.05)	0.22 (±0.04)	0.25 (±0.05)	0.22 (±0.05)
{RF}	DeepConvLSTM	0.25 (±0.06)	0.16 (±0.06)	0.27 (±0.04)	0.18 (±0.04)	0.26 (±0.03)	0.17 (±0.03)

The best performing model for each sensor and validation setup is highlighted in bold.

**Table 4:** Comparison of different cross-validation methods and different performance metrics (column) for different models on two sensor combinations (row).

Sensors	Model	k-fold		leave-recordings-out		leave-one-subject-out	
		Acc	*F*_1_	Acc	*F*_1_	Acc	*F*_1_
{LW, RW}	CNN-LSTM	**0.4** (±0.01)	**0.36** (±0.02)	**0.41** (±0.04)	**0.37** (±0.04)	**0.4** (±0.05)	**0.35** (±0.06)
{LW, RW}	ResNet	0.34 (±0.06)	0.33 (±0.07)	0.35 (±0.02)	0.33 (±0.03)	0.35 (±0.07)	0.33 (±0.06)
{LW, RW}	DeepConvLSTM	0.32 (±0.03)	0.21 (±0.04)	0.27 (±0.06)	0.17 (±0.05)	0.31 (±0.03)	0.19 (±0.02)
{LW, ST}	CNN-LSTM	**0.41** (±0.05)	**0.38** (±0.06)	**0.42** (±0.06)	**0.38** (±0.06)	**0.4** (±0.03)	**0.34** (±0.03)
{LW, ST}	ResNet	0.34 (±0.05)	0.32 (±0.03)	0.37 (±0.05)	0.34 (±0.05)	0.35 (±0.06)	0.33 (±0.05)
{LW, ST}	DeepConvLSTM	0.32 (±0.07)	0.24 (±0.09)	0.32 (±0.05)	0.25 (±0.06)	0.33 (±0.03)	0.25 (±0.04)
{LW, LF}	CNN-LSTM	**0.38** (±0.03)	**0.34** (±0.03)	**0.41** (±0.05)	**0.36** (±0.04)	**0.4** (±0.02)	**0.36** (±0.03)
{LW, LF}	ResNet	0.31 (±0.04)	0.3 (±0.04)	0.33 (±0.04)	0.31 (±0.04)	0.34 (±0.04)	0.32 (±0.03)
{LW, LF}	DeepConvLSTM	0.32 (±0.03)	0.23 (±0.03)	0.29 (±0.04)	0.2 (±0.06)	0.32 (±0.06)	0.23 (±0.06)
{LW, RF}	CNN-LSTM	**0.42** (±0.04)	**0.37** (±0.04)	**0.42** (±0.04)	**0.37** (±0.04)	**0.43** (±0.04)	**0.38** (±0.04)
{LW, RF}	ResNet	0.36 (±0.03)	0.34 (±0.04)	0.33 (±0.04)	0.32 (±0.04)	0.34 (±0.04)	0.33 (±0.05)
{LW, RF}	DeepConvLSTM	0.31 (±0.04)	0.23 (±0.03)	0.3 (±0.06)	0.22 (±0.05)	0.32 (±0.04)	0.24 (±0.06)
{RW, ST}	CNN-LSTM	**0.38** (±0.04)	0.32 (±0.04)	**0.39** (±0.06)	0.32 (±0.05)	**0.38** (±0.03)	**0.32** (±0.04)
{RW, ST}	ResNet	0.36 (±0.08)	0.33 (±0.07)	0.32 (±0.03)	0.32 (±0.04)	0.32 (±0.06)	0.31 (±0.06)
{RW, ST}	DeepConvLSTM	0.31 (±0.05)	0.22 (±0.06)	0.3 (±0.04)	0.22 (±0.04)	0.3 (±0.02)	0.21 (±0.04)
{RW, LF}	CNN-LSTM	**0.39** (±0.07)	**0.34** (±0.07)	**0.38** (±0.04)	**0.31** (±0.03)	**0.39** (±0.06)	**0.33** (±0.05)
{RW, LF}	ResNet	0.32 (±0.03)	0.31 (±0.03)	0.31 (±0.04)	0.3 (±0.04)	0.31 (±0.06)	0.3 (±0.06)
{RW, LF}	DeepConvLSTM	0.32 (±0.05)	0.22 (±0.04)	0.31 (±0.05)	0.21 (±0.05)	0.3 (±0.03)	0.21 (±0.03)
{RW, RF}	CNN-LSTM	**0.39** (±0.06)	**0.33** (±0.06)	**0.39** (±0.02)	**0.33** (±0.03)	**0.38** (±0.05)	**0.32** (±0.05)
{RW, RF}	ResNet	0.34 (±0.07)	0.32 (±0.08)	0.32 (±0.06)	0.31 (±0.07)	0.34 (±0.05)	0.32 (±0.05)
{RW, RF}	DeepConvLSTM	0.3 (±0.04)	0.21 (±0.04)	0.3 (±0.02)	0.21 (±0.02)	0.31 (±0.07)	0.21 (±0.07)
{ST, LF}	CNN-LSTM	**0.33** (±0.05)	**0.28** (±0.05)	**0.32** (±0.03)	**0.26** (±0.02)	**0.32** (±0.05)	**0.27** (±0.05)
{ST, LF}	ResNet	0.27 (±0.03)	0.26 (±0.03)	0.24 (±0.05)	0.23 (±0.05)	0.22 (±0.05)	0.21 (±0.05)
{ST, LF}	DeepConvLSTM	0.31 (±0.06)	0.23 (±0.06)	0.29 (±0.03)	0.21 (±0.03)	0.28 (±0.03)	0.19 (±0.03)
{ST, RF}	CNN-LSTM	**0.34** (±0.07)	**0.28** (±0.08)	**0.34** (±0.08)	**0.28** (±0.09)	**0.34** (±0.01)	**0.27** (±0.02)
{ST, RF}	ResNet	0.25 (±0.04)	0.23 (±0.04)	0.28 (±0.05)	0.26 (±0.06)	0.28 (±0.05)	0.26 (±0.05)
{ST, RF}	DeepConvLSTM	0.29 (±0.03)	0.21 (±0.04)	0.29 (±0.03)	0.21 (±0.05)	0.29 (±0.03)	0.19 (±0.03)
{LF, RF}	CNN-LSTM	**0.33** (±0.04)	**0.27** (±0.05)	**0.33** (±0.04)	**0.28** (±0.04)	**0.31** (±0.03)	**0.26** (±0.02)
{LF, RF}	ResNet	0.22 (±0.06)	0.21 (±0.07)	0.23 (±0.05)	0.22 (±0.05)	0.22 (±0.02)	0.21 (±0.04)
{LF, RF}	DeepConvLSTM	0.28 (±0.04)	0.19 (±0.03)	0.27 (±0.04)	0.19 (±0.05)	0.26 (±0.03)	0.18 (±0.03)

The best performing model for each sensor and validation setup is highlighted in bold.

**Table 5:** Comparison of different cross-validation methods and different performance metrics (column) for different models on three sensor combinations (row).

Sensors	Model	k-fold		leave-recordings-out		leave-one-subject-out	
		Acc	*F*_1_	Acc	*F*_1_	Acc	*F*_1_
{LW, RW, ST}	CNN-LSTM	**0.45** (±0.04)	**0.4** (±0.04)	**0.43** (±0.05)	**0.38** (±0.06)	**0.43** (±0.04)	**0.38** (±0.04)
{LW, RW, ST}	ResNet	0.38 (±0.03)	0.36 (±0.03)	0.36 (±0.04)	0.34 (±0.05)	0.36 (±0.05)	0.35 (±0.04)
{LW, RW, ST}	DeepConvLSTM	0.33 (±0.03)	0.25 (±0.03)	0.34 (±0.03)	0.27 (±0.03)	0.34 (±0.08)	0.25 (±0.1)
{LW, RW, LF}	CNN-LSTM	**0.44** (±0.04)	**0.39** (±0.04)	**0.43** (±0.02)	**0.39** (±0.02)	**0.43** (±0.05)	**0.39** (±0.04)
{LW, RW, LF}	ResNet	0.33 (±0.04)	0.31 (±0.04)	0.38 (±0.05)	0.36 (±0.06)	0.37 (±0.03)	0.36 (±0.02)
{LW, RW, LF}	DeepConvLSTM	0.33 (±0.04)	0.24 (±0.04)	0.32 (±0.03)	0.25 (±0.04)	0.32 (±0.04)	0.24 (±0.07)
{LW, RW, RF}	CNN-LSTM	**0.44** (±0.05)	**0.39** (±0.06)	**0.44** (±0.04)	**0.4** (±0.04)	**0.45** (±0.03)	**0.4** (±0.04)
{LW, RW, RF}	ResNet	0.36 (±0.06)	0.35 (±0.06)	0.36 (±0.04)	0.34 (±0.04)	0.37 (±0.09)	0.35 (±0.1)
{LW, RW, RF}	DeepConvLSTM	0.32 (±0.05)	0.26 (±0.05)	0.34 (±0.05)	0.25 (±0.06)	0.32 (±0.02)	0.24 (±0.04)
{LW, ST, LF}	CNN-LSTM	**0.42** (±0.05)	**0.37** (±0.06)	**0.4** (±0.03)	**0.37** (±0.04)	**0.41** (±0.05)	**0.38** (±0.05)
{LW, ST, LF}	ResNet	0.33 (±0.05)	0.32 (±0.05)	0.3 (±0.01)	0.29 (±0.03)	0.31 (±0.04)	0.3 (±0.03)
{LW, ST, LF}	DeepConvLSTM	0.33 (±0.03)	0.25 (±0.03)	0.32 (±0.05)	0.25 (±0.05)	0.32 (±0.04)	0.24 (±0.05)
{LW, ST, RF}	CNN-LSTM	**0.4** (±0.04)	**0.37** (±0.04)	**0.41** (±0.02)	**0.37** (±0.03)	**0.41** (±0.03)	**0.38** (±0.02)
{LW, ST, RF}	ResNet	0.34 (±0.02)	0.32 (±0.03)	0.32 (±0.04)	0.3 (±0.05)	0.37 (±0.03)	0.35 (±0.05)
{LW, ST, RF}	DeepConvLSTM	0.32 (±0.06)	0.26 (±0.04)	0.29 (±0.04)	0.23 (±0.04)	0.32 (±0.02)	0.25 (±0.02)
{LW, LF, RF}	CNN-LSTM	**0.41** (±0.02)	**0.37** (±0.04)	**0.4** (±0.02)	**0.36** (±0.02)	**0.4** (±0.06)	**0.35** (±0.06)
{LW, LF, RF}	ResNet	0.33 (±0.04)	0.31 (±0.03)	0.33 (±0.03)	0.31 (±0.03)	0.31 (±0.04)	0.29 (±0.04)
{LW, LF, RF}	DeepConvLSTM	0.31 (±0.03)	0.24 (±0.02)	0.31 (±0.04)	0.24 (±0.04)	0.32 (±0.01)	0.24 (±0.02)
{RW, ST, LF}	CNN-LSTM	**0.39** (±0.04)	**0.34** (±0.05)	**0.39** (±0.04)	**0.34** (±0.04)	**0.38** (±0.03)	**0.33** (±0.03)
{RW, ST, LF}	ResNet	0.32 (±0.04)	0.31 (±0.05)	0.31 (±0.07)	0.3 (±0.05)	0.32 (±0.07)	0.31 (±0.06)
{RW, ST, LF}	DeepConvLSTM	0.32 (±0.04)	0.24 (±0.04)	0.32 (±0.05)	0.24 (±0.05)	0.31 (±0.03)	0.23 (±0.02)
{RW, ST, RF}	CNN-LSTM	**0.39** (±0.04)	**0.33** (±0.05)	**0.38** (±0.05)	**0.33** (±0.05)	**0.38** (±0.04)	**0.33** (±0.05)
{RW, ST, RF}	ResNet	0.32 (±0.06)	0.31 (±0.06)	0.31 (±0.04)	0.3 (±0.04)	0.31 (±0.02)	0.29 (±0.02)
{RW, ST, RF}	DeepConvLSTM	0.31 (±0.05)	0.22 (±0.05)	0.31 (±0.03)	0.22 (±0.02)	0.31 (±0.04)	0.23 (±0.05)
{RW, LF, RF}	CNN-LSTM	**0.37** (±0.07)	**0.33** (±0.07)	**0.4** (±0.04)	**0.33** (±0.04)	**0.39** (±0.06)	**0.34** (±0.07)
{RW, LF, RF}	ResNet	0.32 (±0.06)	0.3 (±0.05)	0.29 (±0.05)	0.28 (±0.04)	0.3 (±0.06)	0.29 (±0.06)
{RW, LF, RF}	DeepConvLSTM	0.31 (±0.03)	0.22 (±0.03)	0.29 (±0.05)	0.2 (±0.05)	0.3 (±0.03)	0.21 (±0.02)
{ST, LF, RF}	CNN-LSTM	**0.34** (±0.04)	**0.29** (±0.03)	**0.34** (±0.05)	**0.3** (±0.06)	**0.33** (±0.04)	**0.29** (±0.05)
{ST, LF, RF}	ResNet	0.25 (±0.06)	0.25 (±0.06)	0.25 (±0.05)	0.24 (±0.05)	0.25 (±0.06)	0.24 (±0.06)
{ST, LF, RF}	DeepConvLSTM	0.3 (±0.02)	0.22 (±0.02)	0.29 (±0.04)	0.22 (±0.03)	0.3 (±0.03)	0.21 (±0.04)

The best performing model for each sensor and validation setup is highlighted in bold.

**Table 6:** Comparison of different cross-validation methods and different performance metrics (column) for different models on four sensor combinations (row).

Sensors	Model	k-fold		leave-recordings-out		leave-one-subject-out	
		Acc	*F*_1_	Acc	*F*_1_	Acc	*F*_1_
{LW, RW, ST, LF}	CNN-LSTM	**0.45** (±0.03)	**0.41** (±0.03)	**0.44** (±0.04)	**0.4** (±0.03)	**0.44** (±0.03)	**0.4** (±0.03)
{LW, RW, ST, LF}	ResNet	0.36 (±0.07)	0.35 (±0.08)	0.36 (±0.04)	0.35 (±0.05)	0.36 (±0.05)	0.35 (±0.06)
{LW, RW, ST, LF}	DeepConvLSTM	0.33 (±0.05)	0.27 (±0.06)	0.33 (±0.04)	0.25 (±0.05)	0.35 (±0.05)	0.27 (±0.05)
{LW, RW, ST, RF}	CNN-LSTM	**0.44** (±0.03)	**0.39** (±0.03)	**0.44** (±0.03)	**0.4** (±0.04)	**0.43** (±0.04)	**0.4** (±0.04)
{LW, RW, ST, RF}	ResNet	0.33 (±0.06)	0.32 (±0.07)	0.33 (±0.04)	0.32 (±0.05)	0.35 (±0.05)	0.33 (±0.05)
{LW, RW, ST, RF}	DeepConvLSTM	0.33 (±0.02)	0.26 (±0.02)	0.32 (±0.08)	0.26 (±0.08)	0.35 (±0.02)	0.28 (±0.02)
{LW, RW, LF, RF}	CNN-LSTM	**0.43** (±0.04)	**0.4** (±0.03)	**0.44** (±0.04)	**0.4** (±0.03)	**0.44** (±0.02)	**0.4** (±0.02)
{LW, RW, LF, RF}	ResNet	0.35 (±0.03)	0.34 (±0.05)	0.36 (±0.05)	0.35 (±0.05)	0.36 (±0.05)	0.34 (±0.05)
{LW, RW, LF, RF}	DeepConvLSTM	0.34 (±0.02)	0.27 (±0.03)	0.34 (±0.07)	0.27 (±0.07)	0.35 (±0.05)	0.29 (±0.06)
{LW, ST, LF, RF}	CNN-LSTM	**0.41** (±0.04)	**0.38** (±0.04)	**0.42** (±0.04)	**0.38** (±0.04)	**0.41** (±0.06)	**0.38** (±0.06)
{LW, ST, LF, RF}	ResNet	0.33 (±0.06)	0.32 (±0.06)	0.33 (±0.04)	0.32 (±0.04)	0.3 (±0.05)	0.29 (±0.04)
{LW, ST, LF, RF}	DeepConvLSTM	0.32 (±0.04)	0.26 (±0.03)	0.32 (±0.03)	0.26 (±0.03)	0.32 (±0.05)	0.26 (±0.06)
{RW, ST, LF, RF}	CNN-LSTM	**0.39** (±0.07)	**0.34** (±0.07)	**0.38** (±0.02)	**0.33** (±0.04)	**0.38** (±0.04)	**0.33** (±0.03)
{RW, ST, LF, RF}	ResNet	0.33 (±0.06)	0.31 (±0.06)	0.33 (±0.06)	0.32 (±0.07)	0.29 (±0.01)	0.28 (±0.02)
{RW, ST, LF, RF}	DeepConvLSTM	0.32 (±0.05)	0.24 (±0.08)	0.33 (±0.02)	0.25 (±0.02)	0.3 (±0.04)	0.23 (±0.04)

The best performing model for each sensor and validation setup is highlighted in bold.

**Table 7:** Comparison of different cross-validation methods and different performance metrics (column) for different models on all five sensors combined (row).

Sensors	Model	k-fold		leave-recordings-out		leave-one-subject-out	
		Acc	*F*_1_	Acc	*F*_1_	Acc	*F*_1_
{LW, RW, ST, LF, RF}	CNN-LSTM	**0.44** (±0.06)	**0.4** (±0.06)	**0.41** (±0.02)	**0.38** (±0.03)	**0.43** (±0.07)	**0.38** (±0.06)
{LW, RW, ST, LF, RF}	ResNet	0.35 (±0.03)	0.34 (±0.04)	0.37 (±0.07)	0.36 (±0.08)	0.33 (±0.04)	0.33 (±0.04)
{LW, RW, ST, LF, RF}	DeepConvLSTM	0.34 (±0.04)	0.28 (±0.04)	0.31 (±0.03)	0.24 (±0.05)	0.33 (±0.05)	0.26 (±0.06)

The best performing model for each sensor and validation setup is highlighted in bold.

Across all scenarios (two, three, four, and five sensor combinations), the CNN-LSTM model consistently outperforms the ResNet and DeepConvLSTM models regarding the accuracy and *F*_1_ score for all cross-validation methods. The wrist sensors LW and RW appear to be crucial in achieving better performance, while the addition of the ST (pelvis) sensor further improves the results. Including ankle sensors (LF and RF) shows additional performance gains, however, the improvement diminishes when moving from four to five sensors. In conclusion, the CNN-LSTM model is the most suitable choice for the given problem among the compared models. The combination of wrist, pelvis, and ankle sensors proves beneficial in improving the model’s performance, with wrist sensors being the most critical in providing valuable information. The performance of the deep learning models on the dataset demonstrates its reliability and suitability for activity recognition tasks. The dataset’s fitness for purpose is further evidenced by the consistent improvement in model performance when using combinations of different sensors, which suggests that the dataset effectively captures the relevant information needed for activity recognition. The preprocessed dataset with an adapted folder structure for machine learning applications can be easily accessed through Zenodo, enabling effortless collaboration and sharing of data amongst researchers^13^.

## Usage Notes

The presence of multiple activities in a single recording and the proper ordering of the recording files may prompt researchers to examine the sequential behavior of nurses. Hidden Markov models (HMMs), which include transition probabilities, are a structured probabilistic model that can be used to analyze sequential behavior by forming a probability distribution of sequences.

## Data Availability

The code for the application and previously mentioned models is shared on GitHub (https://github.com/hpi-dhc/sonar).
